# Dental Caries Prevalence in Human Immunodeficiency Virus Infected Patients Receiving Highly Active Anti-Retroviral Therapy in Kermanshah, Iran

**Published:** 2014-02-03

**Authors:** Loghman Rezaei-Soufi, Poorandokht Davoodi, Hamid Reza Abdolsamadi, Mina Jazaeri, Hossein Malekzadeh

**Affiliations:** 1 Department of Operative Dentistry, Dental Research Center, Faculty of Dentistry, Hamadan University of Medical Sciences, Hamadan, Iran; 2 Department of Oral Medicine, Faculty of Dentistry, Hamadan University of Medical Sciences, Hamadan, Iran; 3 Department of Oral Medicine, Faculty of Dentistry, Jundishapour University of Medical Sciences, Ahvaz, Iran

**Keywords:** Dental Caries, HIV Infection, Anti-Retroviral Agents, Root Caries, Iran

## Abstract

**Objective:**

Introduction of new approaches for the treatment of human immunodefi- ciency virus (HIV) infection such as anti-retroviral medicines has resulted in an in- crease in the life expectancy of HIV patient. Evaluating the dental health status as a part of their general health care is needed in order to improve the quality of life in these patients. The aim of this study was to compare the root and crown caries rate in HIV patients receiving highly active antiretroviral therapy (HAART) with that rate in HIV patients without treatment option.

**Materials and Methods:**

This cross sectional study consisting of 100 individuals of both genders with human immunodeficiency virus were divided into two groups: i. group 1 (treat- ment group) including 50 patients with acquired immunodeficiency syndrome (AIDS) receiving HAART and ii. group 2 (control group) including 50 HIV infected patients not receiving HAART. Dental examinations were done by a dentist under suitable light using periodontal probe. For each participant, numbers of decay (D), missed (M), filled (F), Decayed missed and filled teeth (DMFT), decay surface (Ds), missed surface (Ms), filled surface (Fs), Decayed missed and filled surfaces (DMFS), and tooth and root caries were recorded. Data were analyzed using Chi-square test and independent t test using SPSS 13.0, while p-value of <0.05 was considered statistically significant in all analysis.

**Results:**

The mean and standard deviation (SD) of decayed, missed and filled teeth of those who were on highly active antiretroviral therapy was 6.86 ± 3.57, 6.39 ± 6.06 and 1.89 ± 1.93, respectively. There was no significant difference between these values regarding to the treat- ment of patients. The mean and standard deviation of DMFT, DMFS and the number of de- cayed root surfaces were 15.14 ± 6.09, 56.79 ± 28.56, and 4.96 ± 2.89 in patients treated by anti-retroviral medicine which were not significantly different compared to those without this treatment.

**Conclusion:**

According to the results of the present study, highly active antiretroviral ther- apy could not be considered as a single factor for dental caries prevalence in HIV-infected patients. However, more research is recommended to evaluate the cariogenic potential of these medicines.

## Introduction

Acquired immunodeficiency virus disease is a fatal disorder which is characterized by serious opportunistic infections and neoplasms (1). However, with introduction of anti-retroviral drugs, it is now considered a chronic disease, while the infected population is increasing (2). Unfortunately, update reports have shown a rise in human immunodeficiency virus (HIV) infection prevalence despite new medications (3). There are numerous oral lesions associated with HIV infection which are usually found in the early stages of the disease. Hence, it is vital to be able to recognize and to treat common oral diseases including dental caries early in patients with HIV (4).

Saliva has important roles in oral health and prevention of dental caries including clearance effects, buffering capacity, balancing de/ remineralization, antimicrobial properties and production of antibodies (5). Infiltration of human immunodeficiency virus and proliferation of CD8 lymphocytes in salivary glands (6) along with using highly active antiretroviral therapy (HAART) decrease the salivary flow rate and change the normal microbial flora of the oral cavity; therefore, they are considered to be the main factors resulting in dental caries in HIV infected patients (7). Chronic hyposalivation occurring in almost 30-40% of HIV infected patients is another important factor increasing the risk of tooth caries (8). In addition to the factors mentioned above, poor oral hygiene, neglect dental and oral health, periodontal diseases and a diet rich in carbohydrates increase the risk of tooth caries (9). However, the results of a research conducted on the prevalence of root caries in HIV infected patients revealed that root caries did not occur frequently in HIV infected individuals as compared to healthy ones (10). Ponnam et al. (11) evaluated dental caries rate among HIV infected patients on HAART and concluded that these medication has no effect on dental caries prevalence in HIV infected patients. However, Bretz et al. (12) reported that those were treated by anti-retroviral agents had a lower incidence of teeth decay as compared to the patients without treatment option. However, Nittayananta et al. (7) showed that taking these drugs for long term was related to the cervical caries lesions.

Although the previous studies have focused on the periodontal problems and mucosal lesions of the HIV infected population, especially in children (13), there is insufficient data showing the relation between dental caries rate and taking HAART. So, this study was conducted to evaluate the prevalence of teeth caries in patients with AIDS receiving HAART and to compare the prevalence of the root and crown caries with HIV infected patients not receiving HAART.

## Materials and Methods

This cross sectional study included 100 HIV infected individuals of both genders who were under regular medical care in Shahid Ghazi Institute in Kermanshah, Iran, supervised by the World Health Organization. An approval was obtained from Ethics Committee of Hamadan University of Medical Sciences; in addition, a written consent was obtained from all participants after being informed verbally about purpose of the study. They were assured that participation in the study would have no effect on their treatment procedure. All participants had at least three years documented HIV infection and had been receiving HAART for more than a year. Those patients who had history of other diseases, or those who were not under medical care at the Shahid Ghazi Institute were excluded from the study.

Subjects were divided into two groups as follows: i. Group 1 (treatment group) including 50 patients with acquired immunodeficiency syndrome (AIDS) receiving HAART and ii. Group 2 (control group) including 50 HIV infected patients not receiving HAART. Demographic and clinical data including age, gender, use of anti-retroviral medicine, xerostomia (according to its sign and symptoms including no salivary pool), and smoking were documented in a questionnaire for each patients. Dental examination was conducted by a qualified dentist in order to diagnose root and crown caries on a dental chair under suitable light, in a blind manner. Elimination of debris, plaque and calculus prophylaxis and scaling (if needed) were performed before examination of teeth.

Wisdom teeth, teeth extracted due to trauma or orthodontic treatment, and teeth filled because of aesthetic issues were excluded from the study. In order to disclose root caries, gingiva was displaced by gentle air flow, and root surfaces were explored with a hand instrument to find caries lesions. All teeth surfaces were examined by a World Health Organization (WHO) periodontal probe. After dental examination of each participant, numbers of decay (D), missed (M), filled (F), Decayed missed and filled teeth (DMFT), decay surface (Ds), missed surface (Ms), filled surface (Fs), Decayed missed filled surfaces (DMFS) had tooth and root caries were recorded in the questionnaire. Data were analyzed by Chi-square test and independent t test using Statistical Package for the Social Sciences (SPSS), version 13 (SPSS Inc., Chicago, USA), while p value of <0.05 was considered statistically significant in all analyses.

## Results

Demographic data of all participants including age and sex are shown in table 1. The mean age values of treatment and control groups were 36 and 38 years old, respectively. The obtained results showed that 71.4% of Group 1 and 81% of Group 2 were male. In comparing gender and age distribution between Group 1 and 2, no significant differences were found (p>0.05). The result showed that in Group 1, 68% had xerostomia, 81% were smokers, and 95% used drugs other than HAART, presented in table 2, while the results of chi-square showed no significant difference among the prevalence of these factors between the two groups. Table 3 illustrates the numbers of decay (D), missed (M), filled (F), DMFT, decay surface (Ds), missed surface (Ms), filled surface (Fs), DMFS, tooth and root caries, and p values. According to the results of the present study, there was no significant difference between the two groups regarding these factors (p>0.05).

**Table 1:** Comparison of demographic data between treatment (with HARRT) and control (without HAART) groups.


Groups		No.		Age range (yrs)		Mean age (yrs)		Gender (%)	
				

**Treatment**			22	26-51	36		M:71.4/F:28.6	
				
**Control**			28	27-54	38		M: 81.8/F:18.2	
				


**Table 2:** Comparison prevalence of Xerostomia, smoking and drugs except HAART between treatment (with HARRT) and control (without HARRT) using Chi-square test.


	Groups	No.	P value*
			

**Xerostomia**	Treatment	15	0.295
	Control	7	
**Smoking**	Treatment	18	0.320
	Control	17	
**Other drugs**	Treatment	21	0.852
	Control	17	


*; P value of p<0.05 was considered significant.

**Table 3:** Comparison of Mean ± SD of decay (D), missed (M), filled (F), DMFT, decay surface (Ds), missed surface (Ms), filled surface (Fs), DMFS, and root caries between treatment (with HARRT) and control (without HARRT) using independent t test.


Variable	Groups	Mean ± SD	P value*

**D**	Treatment	6.86 ± 3.57	0.16
	Control	5.36 ± 3.77	
**M**	Treatment	6.39 ± 6.06	0.26
	Control	8.14 ± 4.4	
**F**	Treatment	1.89 ± 1.93	0.14
	Control	0.95 ± 1.09	
**DMFT**	Treatment	15.14 ± 6.09	0.67
	Control	14.45 ± 5.05	
**Ds**	Treatment	21.25 ± 14.09	0.52
	Control	18.73 ± 11.19	
**Ms**	Treatment	29.57 ± 29.04	0.41
	Control	35.55 ± 19.9	
**Fs**	Treatment	5.96 ± 6.08	0.08
	Control	3.41 ± 2.25	
**DMFS**	Treatment	56.79 ± 28.56	0.90
	Control	57.68 ± 18.76	
** Root caries**	Treatment	4.96 ± 2.89	0.13
	Control	3.77 ± 2.49	
**Percentage of tooth caries**	Treatment	34.52 ± 2.03	0.21
	Control	27.42 ± 1.9	
**Percentage of root caries**	Treatment	26.56 ± 2.11	0.07
	Control	18.29 ± 1.03	


*; P value of p<0.05 was considered significant.

## Discussion

Xerostomia is a common manifestation of HIV infection which occurs as a consequence of salivary gland hypofunction due to the infection itself or the medicine used for the treatment (14). HAART, a new approach for AIDS treatment, results in dental caries through two different routes. First, it influences salivary glands function and declines salivary flow rate (15), and second, it contains the high concentration of glucose (16).

In the current study, in order to reduce any biases in the obtained results, factors which probably influence the rate of tooth caries such as xerostomia, smoking, drugs like methadone, age and gender were compared using chi-square test. Since the awareness and knowledge to seek treatment for oral pathologies such as dental caries are different among those infected with HIV, participants enrolled in the present study were selected from one special care center for HIV-infected patients. The results showed that dental caries prevalence assessed by different indicators including DMFT, DMFS, and root caries was not significantly different between treatment and control groups. However, the mean value of DMFT, the average number of decayed roots, and the mean percentage of root and crown caries were higher in treatment group.

Similar to our finding, Ponnam et al. (11) found that the prevalence of dental caries in HIV-infected children did not differ significantly in regard to the treatment with HAART. The outcome of their study showed that although many HIV-infected children experienced rampant and severe caries rate, therapeutic agent caused no significant trend in severity and prevalence of tooth caries. Rwenyonyi et al. (17) also found no significant difference between dental caries rate in relation to treatment with HAART.

In contrast to the present results, studies by Bretz et al. (12), Nittayananta et al. (7) and Glick et al. (18) showed that there was a relationship between HAART and dental caries rate. Nittoyananta et al. (7) concluded that higher prevalence of dental caries occurred in long term use of antiretroviral medication; however, short term use of these agents had no effect on the teeth decay. According to their study, patients who received HAART, especially for a short term, experienced higher rate of xerostomia and periodontal pocket, but the incidence of cervical decay did not show much difference. Bretz et al. (12) showed that dental caries prevalence was lower in patients treated by HAART. The results of their study suggested that although the salivary flow rate of patients treated with HAART was reduced, they showed lower rates of dental caries as compared to those who received no treatment. In contrast to these studies, Glick et al. (18) revealed that an increase in rate of dental caries was associated with the HAART.

Regarding to the limitation of the present study including different duration of the infection, receiving the HAART for varies period, no opportunity for radiographic study and examination by only one dentist, more researches on this topic is highly recommended.

## Conclusion

According to the results of the present study, HAART could not be considered as a single factor in dental caries prevalence in HIV-infected patients in Kermanshah, Iran. However, more research is recommended to evaluate the cariogenic potential of these medications.
